# Analysis of the Weight Management Behavior of Chinese Pregnant Women: An Integration of the Protection Motivation Theory and the Information-Motivation-Behavioral Skills Model

**DOI:** 10.3389/fpubh.2022.759946

**Published:** 2022-02-04

**Authors:** Jinjin Ge, Shiqi Zhao, Xueqing Peng, Anita Nyarkoa Walker, Ni Yang, Hua Zhou, Li Wang, Chi Zhang, Meng Zhou, Hua You

**Affiliations:** ^1^Department of Social Medicine and Health Education, School of Public Health, Nanjing Medical University, Nanjing, China; ^2^Department of Gynecology and Obstetrics, Changzhou Maternal and Child Health Care Hospital Affiliated to Nanjing Medical University, Changzhou, China; ^3^School of Nursing, Nanjing Medical University, Nanjing, China; ^4^Institute of Healthy Jiangsu Development, Nanjing Medical University, Nanjing, China

**Keywords:** pregnancy, weight management, protection motivation theory, information-motivation-behavioral skills model, integrative theoretical model

## Abstract

Inappropriate gestational weight gain has become a public health concern that threatens maternal and child health. Pregnant women's ability to manage their weight during pregnancy directly impacts their weight gain. In this study, we integrated the protection motivation theory and the information-motivation-behavioral skills model to develop an integrative theoretical model suitable for pregnancy weight management and reveal significant explainable factors of weight management behaviors during pregnancy. Based on a cross-sectional survey of 550 pregnant women from Jiangsu province, we came up with our findings. The results showed that several factors influenced pregnancy weight management behavior. According to the research, information, self-efficacy, response costs, and behavioral skills were significantly associated with weight management behaviors during pregnancy, while behavioral skills were also significant mediators of information, self-efficacy, and behavior. Furthermore, the information related to pregnancy weight management had the biggest impact on weight management behavior during pregnancy. The results of the model fit were acceptable and the integrative model could explain 30.6% of the variance of weight management behavior during pregnancy, which implies that the integrative theoretical model can effectively explain and predict weight management behaviors during pregnancy. Our study provides practical implications for the integrative model in improving pregnancy weight management behavior and offers a theoretical base for the weight management of pregnant women.

## Introduction

Having an appropriate gestational weight gain (GWG) is critical for fetal growth and development, while both low and high GWG pose severe health risks to the mother and child. Inappropriate GWG not only has short-term health effects such as causing pregnancy complications, cesarean deliveries, macrosomia, and preterm birth, but also long-term health effects such as postpartum weight retention and childhood chronic diseases (1–4). According to recommendations made by the Institute of Medicine (IOM) (5), appropriate GWG is as low as 40%, and excessive GWG has exceeded 50% in some countries (6, 7). Therefore, improving weight management during pregnancy has become a pressing concern today.

Pregnancy weight management, as a protective behavior of pregnant women to promote maternal and infant health, the formation process and internal influencing factors of it should be clarified first to better promote behavior change. Based on a behavioral perspective at the individual level, the formation of weight management behavior during pregnancy is influenced by information, psychological perceptions, and behavioral skills. Several studies have shown that pregnant women are unaware of the serious consequences of excessive GWG because of a lack of correct knowledge about pregnancy weight management (8, 9). Women who perceive the advantages of weight management during pregnancy are unable to act because of a lack of appropriate knowledge (10). At the same time, misunderstanding or exaggerating the negative impact of weight management during pregnancy can also prevent pregnant women from taking action (11). Additionally, the lack of self-management ability is also the reason why obese and overweight pregnant women are unable to develop better self-management skills compared with normal-weight pregnant women (12). Therefore, to understand the formation mechanism of weight management behavior more thoroughly, we need to explore both the internal factors that influence this behavior and the interaction of these factors simultaneously.

Behavioral interventions based on corresponding theories can effectively alter behavior, and behavioral theories derived from psychological and social perspectives play an extremely important role in understanding healthy behavior (13, 14). Based on the successful application of the behavioral theory in other healthy behavior fields, researchers have also begun to apply the healthy behavior theory to the study of pregnancy weight management. Rather than focusing on the complexity of the internal factors in individual behavior formation, most of these studies have explored environmental and individual factors from a macro perspective (15, 16). Some studies focus more on the role of individual perception in behaviors related to pregnancy weight management, ignoring the influence of other factors such as behavioral skills on behavior (17–19). Additionally, some theories focus more on the stage change process of behavior and are often applied to the development of behavioral interventions for pregnancy weight gain, lacking their application in the exploration of factors influencing pregnancy weight management behavior (20). We, therefore, need a more comprehensive theoretical model that will be suitable for explaining weight management behavior during pregnancy from the individual level

Protection motivation theory (PMT) developed by Rogers (21), explains the process of individual behavior change from the perspective of psychological perception. Individuals create behavioral intentions through comprehensive consideration of threat appraisal and coping appraisal to promote behavior change. The threat appraisal refers to the perceived severity and vulnerability of risk factors; the coping appraisal refers to the individual's perception of their ability to deal with risk factors, including response efficacy, self-efficacy, and response costs. An individual's perception of severity, vulnerability, response efficacy, and self-efficacy can promote behavior change, while response costs can weaken behavior change willingness. A study of factors influencing maternal nutritional intake during pregnancy in low- and middle-income countries based on the PMT framework showed that perceived threat of adverse health outcomes could affect dietary behavior during pregnancy (18). Additionally, the application of information intervention based on the PMT framework to the study of pregnant women's willingness to exercise can effectively improve pregnant women's perception of the factors of the PMT and exercise motivation, and encourage women to exercise during pregnancy (22). So, we believe that pregnant women can choose the most appropriate weight management behavior if they are aware of the hazards of inappropriate GWG, realize how vulnerable they are to it, judge the benefits and costs of weight management during pregnancy, and comprehend how to do it appropriately.

Fisher proposed the information-motivation-behavioral skills (IMB) model to guide individuals toward behavior change, with four core determinants: information, motivation, behavioral skills, and behavior (23). The IMB model emphasizes that it is not enough to explain and predict behaviors by motivation dimension alone, the impact of information and behavioral skills on health behaviors should also be considered. Based on a variety of factors that may affect behavior change and their complex interactions, the model had been successful in explaining and predicting health behaviors (24, 25). In addition to the motivation factors mentioned above, previous studies have shown that accurate knowledge about weight gain during pregnancy also promotes appropriate GWG (26), suggesting the importance of accurate and reliable information for weight management behaviors during pregnancy. Information and motivation dimensions can directly promote the transformation of health behaviors, but sometimes only these two are not enough to change complex behaviors, and behavioral skills which can directly achieve behavior change are also needed (27). At the same time, individuals who have adequate information and sufficient motivation will actively improve behavioral skills and further promote behavioral change (28). Therefore, it is necessary to have relevant knowledge, sufficient motivation, and corresponding behavioral skills at the same time, to achieve a better change in weight management behaviors during pregnancy.

**Figure 1:**
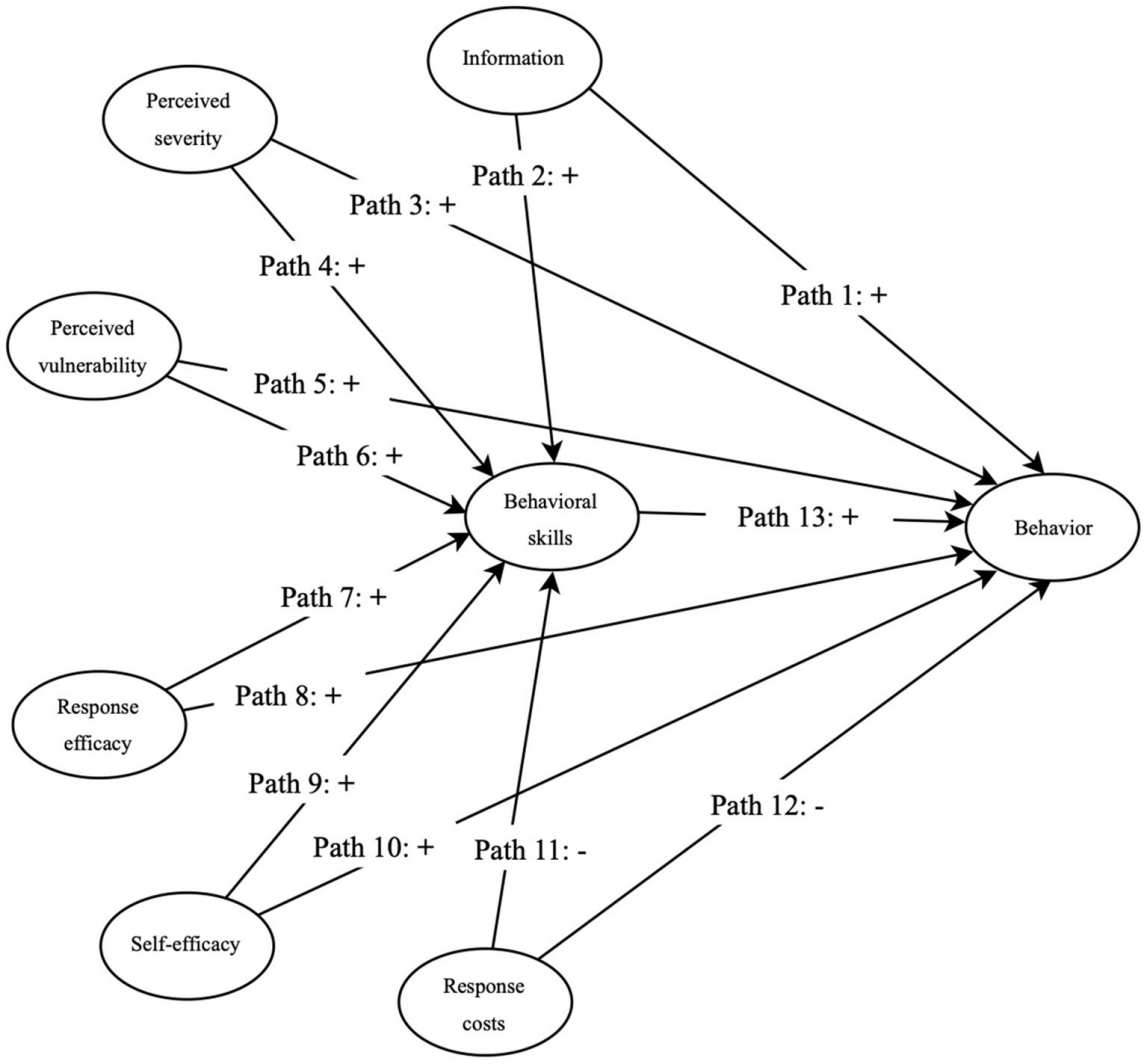
The PMT-IMB model. Behavior, behavior during pregnancy.

Promoting the change of healthy behaviors is the goal of the PMT and the IMB model, and motivation is a key factor for both. To interpret and predict weight management behaviors during pregnancy more comprehensively, the PMT and the IMB model can be integrated into a new model using motivation as the mediating core: the PMT-IMB model (Figure 1). In the previous study on the health management of patients with type 2 diabetes, interventions based on the PMT and the IMB model had achieved good results, but the study did not propose an integrative framework of the two theories (29). The PMT-IMB model replaced the motivation dimensions in the IMB model with five dimensions that describe the formation of motivation in the PMT. These five dimensions also can have a direct or indirect effect on individual behavior through behavioral skills. The path relationships assumed in this study as shown in Figure 1. First, information, perceived severity, perceived vulnerability, response efficacy, self-efficacy, and response costs have direct effects on behavioral skills and weight management behaviors during pregnancy, while behavioral skills also have a direct effect on pregnancy weight management behaviors. Second, information, perceived severity, perceived vulnerability, response efficiency, self-efficacy, and response costs can also have indirect effects on weight management behaviors during pregnancy through behavioral skills. Based on the PMT-IMB model, the purpose of this study is to explore the factors that influence pregnancy weight management behavior and construct an integrative, theoretical model that is appropriate for explaining such behavior.

## Materials and Methods

### Study Setting and Participants

A cross-sectional study was conducted in Changzhou, one of the central cities in the Yangtze River Delta region of China. The study setting was Changzhou Maternal and Child Health Care Hospital, which undertook about 40% of the city's annual deliveries, and could meet the sample requirements of this study. From September to October 2020, pregnant women who visited the obstetrics and gynecology clinic at the institution were enrolled. Inclusion criteria: gestational age ≥ 14 weeks; able to understand and write; and willing to participate in the study. Exclusion criteria: Under 18 years of age; multiple pregnancies; history of psychotic disorder or major depressive episode; history of cardiovascular, hepatic, and renal diseases; history of essential hypertension and diabetes mellitus; other disabilities such as deafness and dumbness.

### Data Collection

The ethical review committee of Nanjing Medical University approved this study before its initiation. We distributed 550 questionnaires to the participants after obtaining informed consent from them, and we received 525 valid returns (95.5%). Each questionnaire took about 25 min to complete.

### Questionnaires

We collected demographic data includes age, height, pre-pregnancy weight, educational level, work status, and household registration.

The self-perception of pregnant women on weight management behaviors during pregnancy was measured using a self-made scale based on the PMT-IMB model. This scale has 31 items across seven dimensions: information (5 items, e.g., “I have knowledge about the benefits of pregnancy weight management for maternal and fetal outcomes”), behavioral skills (5 items, e.g., “I know how to assess whether my weight gain during pregnancy is reasonable”), perceived severity (4 items, e.g., “Abnormal weight during pregnancy can cause chronic postpartum disease and affect my health.”), perceived vulnerability (2 items, e.g., “I think my GWG is normal and there is no need for weight management”), response efficacy (5 items, e.g., “If I manage my weight during pregnancy, my risk of adverse pregnancy outcome will be reduced”), self-efficacy (5 items, e.g., “I believe that I can overcome the obstacles and difficulties associated with pregnancy weight management”), response costs (5 items, e.g., “Weight management during pregnancy requires exercise, which brings trouble to me”). This scale was assessed using a 5-point Likert scoring method, from 1 (strongly disagree) to 5 (strongly agree). A total score of 5 to 25 was achieved for all dimensions except for the perceived severity dimension (ranged from 4 to 20) and vulnerability dimension (ranged from 2 to 10). The Cronbach's alpha of those dimensions ranged from 0.736 to 0.903, the total reliability of the scale was 0.850, the Kaiser–Meyer–Olkin (KMO) was 0.877, the Bartlett's test of sphericity was significant (9713.699, *P* < 0.001) and the total variance of the seven factors was 69.5%. Those all indicated the results of this scale were acceptable.

Weight management behaviors during pregnancy were measured by the adapted Pregnancy Weight Management Strategy Scale (PWMSS) (30), which consisted of four dimensions: management objectives, diet management, exercise management, and self-monitoring regulation, with a total of 18 items. Ratings are based on a five-point Likert scale ranging between 1 (never occurs) and 5 (always occurs), with scores ranging from 18 to 90. The PWMSS was originally developed by Yan et al. (30) have been proved to be reliable and valid. The value of Cronbach's alpha ranged from 0.613 to 0.863, the total reliability of the modified scale was 0.834. Pregnant women were required to choose the most appropriate option based on their situation during the past month. Pregnant women with a higher score adopted more weight management strategies during pregnancy, thus resulting in better weight management outcomes.

### Data Analysis

Statistical significance was defined as a two-sided test *P* < 0.05 in all analyses. After data entry and checking, SPSS 25.0 was used for statistical description and analysis. Continuous variables were described by mean ± standard deviation (SD) or median (interquartile range, IQR); categorical variables were described by frequency and percentage. Reliability analysis and factor analysis were used to test the reliability and validity of scales.

Structural equation modeling has been used widely in model revision, model evaluation, and verification of theoretical development (31). Since this study belongs to the exploration of integrative model and the data collected was non-normally distributed, SmartPLS3.3.3 software was used for data analysis, and partial least squares structural equation modeling was used to test the relationship between each latent variable (32). To optimize the model structure, the PWMSS was dimensionality reduced and the mean value of each dimension was used to represent the behavior of pregnant women in this dimension.

As this was an exploratory study, factor loadings > 0.4 and significant were acceptable (32). Cronbach's alpha (CA) (>0.7) and the compositional reliability (CR) (>0.6) were used to evaluate the internal consistency of the latent variables in the measurement model (33). The average variance extracted (AVE) (>0.5) was used to assessed the convergent validity of the constructs (33).The square root of the AVE of each construct was greater than the correlation coefficient of the construct with any other constructs and the Heterotrait-Monotrait Ratio (HTMT) was <0.85, suggesting good discriminant validity across the constructs (33, 34). Then the bootstrapping algorithm with 5,000 sampling times was set to calculate the significance of the path coefficients (35). *R*^2^ (cut-off points of 0.190, 0.333, and 0.67 represented weak, medium, and large, respectively) and Q^2^ (>0) were used to test the explanatory and predictive power of the model (33). In addition, we tested the fitness of the model using the goodness-of-fit (GoF) value (cut-offs of 0.1, 0.25, 0.36, respectively, for weak, medium, and strong) (36), the standardized root mean square residual (SRMR) (<0.08) and the normed fit index (NFI) (>0.9) (37).

## Results

### Descriptive Statistics

The median age of the participants was 29 years old. The proportion of rural population (58.3%) was slightly higher than that of urban population (41.7%). The majority of pregnant women (66.5%) had a junior college degree or higher, and most of them worked full-time (59.2%). According to the World Health Organization body mass index (BMI) classification criteria, most of the participants were at a normal weight before pregnancy (74.9%). And the median pre-pregnancy BMI was 22.0 (20.0, 24.0). Table 1 displays the demographic characteristics of study participants. The scores of the PWMSS and subscales of the PMT-IMB model were shown in Table 2.

**Table 1:** Demographic characteristic of participants (*n* = 525).

**Variables**		**Median (IQR) /** ***N*** **(%)**
Age		29 (26, 32)
	< 25	55 (10.5%)
	25–29	233 (44.4%)
	30–34	182 (34.7%)
	≥35	55 (10.5%)
Household registration	Rural	306 (58.3%)
	City	219 (41.7%)
Educational level	Junior high school and below	103 (19.6%)
	High school or technical secondary school	73 (13.9%)
	Junior college	140 (26.7%)
	University and above	209 (39.8%)
Work status	Full-time	311 (59.2%)
	No or part-time	214 (40.8%)
Pre-pregnancy BMI		22.0 (20.0, 24.0)
	<18.5 (underweight)	45 (8.6%)
	18.5–24.9 (normal weight)	393 (74.9%)
	25.0–29.9 (overweight)	66 (12.6%)
	≥30.0 (obese)	21 (4%)

*BMI, body mass index*.

**Table 2:** Mean, standard deviation (SD), and range of scores for the constructs of the PMT-IMB model and PWMSS (*n* = 525).

**Variables**	**Mean (SD)**	**Range**
Information	3.0 (0.7)	1–5
Behavioral skills	3.3 (0.8)	1–5
Perceived severity	4.5 (0.7)	1–5
Perceived vulnerability	3.3 (1.1)	1–5
Response efficacy	4.4 (0.7)	1–5
Self-efficacy	3.8 (0.9)	1–5
Response costs	2.6 (0.9)	1–5
PWMSS	49.9 (11.8)	18–90

*PWMSS, Pregnancy Weight Management Strategy Scale*.

### Measurement Model

The values of all factor loadings were higher than 0.65 in Table 3. The Cronbach's alpha for all measurement models ranged from 0.736 to 0.903, the compositional reliability ranged from 0.837 to 0.928, and the AVE of each construct ranged from 0.563 to 0.788. Since all the values were higher than the recommended thresholds, the internal consistency and convergence validity of the measurement model were proved to be good. The square root of the AVE (value on the diagonal) was greater than the correlation coefficient in the same row or columns (Table 4), while the HTMT was lower than 0.7 for all constructs (Table 5), indicating a favorable discriminant validity. Based on these results, the measurement model demonstrated satisfactory reliability and validity.

**Table 3:** Factor loading, internal consistency, and convergent validity of the measurement model.

**Variable**	**Indicator**	**Factor loading**	**Cronbach's alpha**	**CR**	**AVE**
Behavior (BE)	Management objectives	0.782	0.739	0.837	0.563
	Exercise management	0.717			
	Diet management	0.658			
	Self-monitoring regulation	0.833			
Behavioral skills (BS)	BS1	0.775	0.841	0.887	0.614
	BS2	0.830			
	BS3	0.860			
	BS4	0.779			
	BS5	0.658			
Information (IN)	IN1	0.797	0.832	0.883	0.602
	IN2	0.801			
	IN3	0.820			
	IN4	0.796			
	IN5	0.653			
Perceived severity (PS)	PS1	0.899	0.886	0.921	0.746
	PS2	0.923			
	PS3	0.865			
	PS4	0.758			
Perceived vulnerability (PV)	PV1	0.920	0.736	0.881	0.788
	PV2	0.854			
Response efficacy (RE)	RE1	0.834	0.882	0.914	0.681
	RE2	0.872			
	RE3	0.848			
	RE4	0.836			
	RE5	0.728			
Self-efficacy (SE)	SE1	0.786	0.903	0.928	0.721
	SE2	0.852			
	SE3	0.877			
	SE4	0.860			
	SE5	0.868			
Response costs (RC)	RC1	0.740	0.891	0.920	0.699
	RC2	0.865			
	RC3	0.885			
	RC4	0.802			
	RC5	0.880			

*Behavior, weight management behavior during pregnancy*.

**Table 4:** Discriminant validity: Fornell–Larcker criterion.

	**BE**	**IN**	**RC**	**RE**	**SE**	**PS**	**BS**	**PV**
BE	0.751							
IN	0.458	0.776						
RC	−0.304	−0.135	0.836					
RE	0.246	0.252	−0.088	0.825				
SE	0.327	0.255	−0.337	0.398	0.849			
PS	0.215	0.209	−0.102	0.615	0.285	0.864		
BS	0.428	0.529	−0.233	0.321	0.443	0.259	0.783	
PV	0.075	0.07	−0.135	0.033	−0.113	−0.015	−0.051	0.888

*BE, weight management behavior during pregnancy; IN, information; RC, response costs; RE, response efficacy; SE, self-efficacy; PS, perceived severity; BS, behavioral skills; PV, perceived vulnerability*.

**Table 5:** Discriminant validity : Heterotrait-Monotrait Ratio.

	**BE**	**IN**	**RC**	**RE**	**SE**	**PS**	**BS**	**PV**
BE								
IN	0.577							
RC	0.378	0.156						
RE	0.305	0.295	0.112					
SE	0.397	0.295	0.370	0.452				
PS	0.261	0.240	0.131	0.688	0.318			
BS	0.537	0.628	0.266	0.367	0.503	0.301		
PV	0.103	0.098	0.171	0.060	0.137	0.059	0.076	

*BE, weight management behavior during pregnancy; IN, information; RC, response costs; RE, response efficacy; SE, self-efficacy; PS, perceived severity; BS, behavioral skills; PV, perceived vulnerability*.

### Structural Model

The model explained 30.6% of the variance of weight management behavior during pregnancy (*R*^2^ = 0.306) and the Q^2^ for all dependent variables was >0, indicating that the model had good explanatory and predictive ability (Table 6). The value of GoF was 0.415, SRMR was 0.055 and NFI was 0.8. Except for the value of NFI, the values of GoF and SRMR have reached the recommended standards.

**Table 6:** Results of GoF, R^2^, Q^2^.

	**GoF**	***R*** **^2^**	**Q^2^**
Behavior	0.415	0.306	0.170
Behavioral skills	0.491	0.392	0.238

*GoF, goodness of fit; Behavior, weight management behavior during pregnancy*.

**Figure 2:**
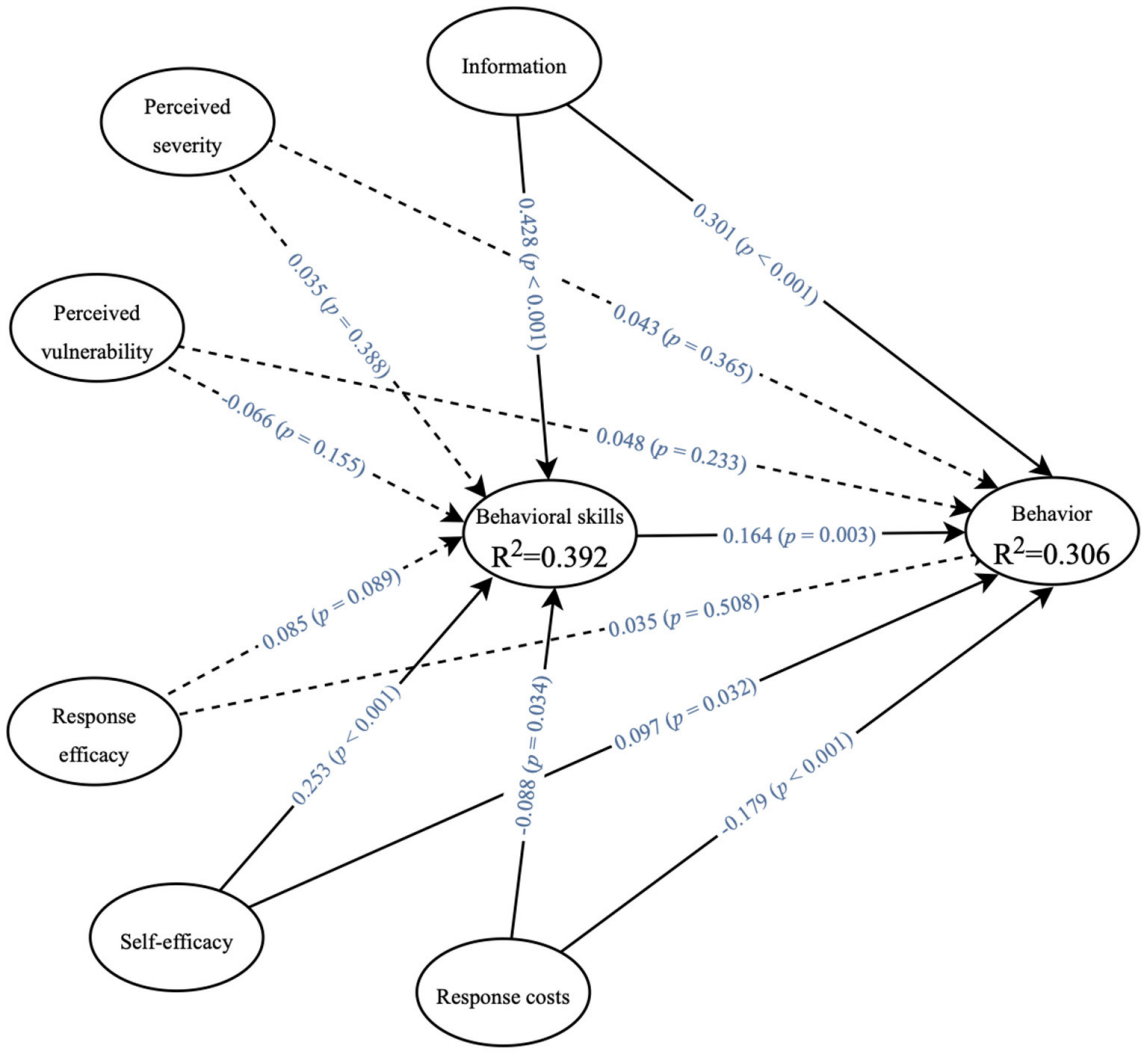
Significance testing results of the structural model path coefficient. Behavior, behavior during pregnancy; Dotted paths indicated non-significant coefficients.

The path analysis results of the structural model were shown in Table 7, Figure 2. The information had a direct positive effect on behavioral skills (β = 0.428, *P* < 0.001) and pregnancy weight management behavior (β = 0.301, *P* < 0.001), and it can also have an indirect positive effect on pregnancy weight management behavior through behavioral skills (β = 0.070, *P* = 0.005). The level of self-efficacy was also directly associated with behavioral skills (β = 0.253, *P* < 0.001) and pregnancy weight management behavior (β = 0.097, *P* = 0.032), and it can also have an indirect positive effect on pregnancy weight management behavior through behavioral skills (β = 0.042, *P* = 0.008). There also had a positive correlation between behavioral skills and weight management behaviors during pregnancy (β = 0.164, *P* = 0.003). At the same time, the response costs had a direct negative effect on behavioral skills (β = −0.088, *P* = 0.034) and weight management behavior during pregnancy (β = −0.179, *P* < 0.001). Nevertheless, the path coefficients of perceived severity, vulnerability, response efficacy with behavioral skills and weight management behaviors during pregnancy did not reach significance.

**Table 7:** The direct, indirect, and total effects among the latent variables in the structural model.

**Variables**	**Behavioral skills**			**Behavior**		
	**Direct effects**	**Indirect effects**	**Total effects**	**Direct effects**	**Indirect effects**	**Total effects**
Information	0.428^**^	-	0.428^**^	0.301^**^	0.070^**^	0.371^**^
Perceived severity	0.035	-	0.035	0.043	0.006	0.049
Perceived vulnerability	−0.066	-	−0.066	0.048	−0.011	0.038
Response efficacy	0.085	-	0.085	0.035	0.014	0.049
Self-efficacy	0.253^**^	-	0.253^**^	0.097^*^	0.042^**^	0.138^**^
Response costs	−0.088^*^	-	−0.088^*^	−0.179^**^	−0.014	−0.193^**^
Behavioral skills	-	-	-	0.164^**^	-	0.164^**^

*Behavior, weight management behavior during pregnancy*;

* *p < 0.05*,

** *p < 0.01*.

## Discussion

The overall findings supported that the integrative model had a good explanatory ability for weight management behaviors during pregnancy.

According to the results of this study, highly relevant information about pregnancy weight management had a direct positive impact on weight management behavior during pregnancy, which was consistent with the findings of other protective behavior studies (23). By comparing the path coefficients of each dimension, the information was found to be the strongest predictor of weight management behavior during pregnancy, which suggested that we should attach great importance to the impact of health-related information on the weight management behavior of pregnant women when carrying out weight management interventions during pregnancy. A previous study has shown that the exercise and weight gain of pregnant women during the COVID-19 pandemic is not optimistic, and improving the knowledge of pregnant women was beneficial to weight management during pregnancy (38). Since some pregnant women still lack the correct knowledge related to pregnancy weight management (39), we should strongly advocate for providing some correct and easy-to-understand information about weight management during pregnancy in health education.

Behavioral skills, which are practical skills based on self-efficacy, have a good explanation and prediction capabilities for a variety of health behaviors. In the integrative model, behavioral skills had a direct positive effect on weight management behavior during pregnancy. According to surveys of young pregnant women, even though they had sufficient knowledge of health behavior, it was still difficult to manage weight during pregnancy due to the lack of knowledge of behavioral skills (40). All of this reminded us that providing pregnant women with pregnancy weight management knowledge is not sufficient, useful methods and practical skills of weight management must also be provided. Additionally, behavioral skills also played an important mediating role between information, self-efficacy, and weight management behavior during pregnancy, which confirmed the original research hypothesis.

As expected, the self-efficacy of pregnant women impacted their weight management behaviors. In this study, self-efficacy refers to the confidence of pregnant women in their ability to successfully manage their weight during pregnancy and maintain an appropriate gestational weight gain. Pregnant women can have a high degree of self-evaluation after realize they can manage their weight well while pregnant, which will promote their weight management behavior. Furthermore, several studies have shown that self-efficacy is not only a significant explanatory factor of protective behaviors, it is also a long-term predictor of behavior change (41–43). Not only did this draw our attention to the predictive ability of self-efficacy in weight management behaviors during pregnancy, but also suggested we should improve the self-efficacy of pregnant women for weight management in future interventions.

Additionally, pregnant women's perceived weight management costs negatively impacted their weight management behavior during pregnancy, and the ability of response costs to explain this behavior was second only to information. According to this study, the higher the response costs perceived by pregnant women, the more difficult it is for them to change their weight management behavior, which was in line with a previous result (11). To promote behavioral changes in pregnant women, we should not only reduce the social and economic costs pregnant women incur for weight management in the real world, but also reduce the costs that pregnant women believe to pay at the cognitive level.

However, the assumptions between perceived severity, perceived vulnerability, response efficacy, and weight management behavior during pregnancy were not valid, which means that the effects of perceived severity, perceived vulnerability, and response efficacy on weight management behavior during pregnancy were not significant. Researchers had observed similar results in previous studies on protective sexual behavior (41). This might be because pregnant women not being aware of their vulnerability to excessive GWG (26). Even if they are aware of the serious consequences of inappropriate GWG, it is still difficult for them to have corresponding action changes. It was also consistent with the results from Table 2, with relatively high perceived severity 4.5 (0.7) and response efficacy 4.4 (0.7) scores, and relatively low perceived vulnerability 3.3 (1.1). These results indicated that multiple dimensions better explain weight management behavior during pregnancy compared to single dimensions, and further proved that the integrative theoretical model provided a more comprehensive explanation of weight management behavior during pregnancy from the individual level. As a result, when undertaking interventions to help pregnant women manage their weight, we should consider their needs from a more scientific and systematic perspective.

## Limitation

We were able to gain a more comprehensive understanding of pregnancy weight management behavior by integrating two theories (i.e., PMT and the IMB model). There are, however, some limitations in this study. Since this study used a self-administered questionnaire to investigate the situation of pregnant women in the past month, it inevitably led to some information bias. Because this study involved only pregnant women in a particular region, the generalizability of the model was limited, and further studies for a wider population are needed. In the results, the value of NFI, one of the model fit indexes, did not meet the recommended standard. This might be because this index is unstable and vulnerable to the complexity of the model (44). However, all of the other correlation indexes have reached the requirements, indicating that the overall results of this study were satisfactory enough. Path analysis revealed that not all dimensions had significant effects on weight management behavior during pregnancy. Perhaps this is because the PMT-IMB model still needs to be tested across different subgroups of the target populations since the study is only a cross-sectional survey.

## Conclusion

The study was the first to integrate two behavior theories, the PMT and the IMB, into a new one that explored how weight management behaviors during pregnancy are influenced. Through a cross-sectional survey, this study systematically analyzed the internal factors influencing weight management behaviors, constructed an integrative theoretical model applicable to pregnancy weight management behaviors, and provided a preliminary theoretical basis for rationally applied this integrative model to pregnancy weight management interventions. We can conclude from the study that: To promote changes in weight management behavior during pregnancy, we should first provide pregnant women with sufficient weight management information, assist them with useful weight management skills, improve the self-efficacy of pregnant women to manage their weights, and reduce the impact of the cost of weight management during pregnancy on pregnant women. Additionally, we must not ignore the role of perceived severity, vulnerability, and response efficacy to cope with weight gain during pregnancy. A longitudinal study can be conducted next to prove the causal relationship between the factors of the PMT-IMB model and weight management behavior during pregnancy and to further prove the applicability of the PMT-IMB model in the explanation, prediction, and intervention of weight management behavior during pregnancy, thus providing a suitable theoretical basis for the formulation of policy programs in this area.

## Data Availability Statement

The raw data supporting the conclusions of this article will be made available by the authors, without undue reservation.

## Ethics Statement

The studies involving human participants were reviewed and approved by the Ethics Committee of Nanjing Medical University. The patients/participants provided their written informed consent to participate in this study.

## Author Contributions

XP, CZ, and HY designed research. JG, SZ, XP, AW, NY, HZ, LW, CZ, MZ, and HY performed research. JG and SZ analyzed data. JG, SZ, and HY wrote the paper. All authors read and approved the final manuscript.

## Funding

This work was supported by National Natural Science Foundation of China (No. 72074122) and Jiangsu Maternal and Child Health Research Project (No. F202028). The funding agency had no role in conducting the study or writing the manuscript.

## Conflict of Interest

The authors declare that the research was conducted in the absence of any commercial or financial relationships that could be construed as a potential conflict of interest.

## Publisher's Note

All claims expressed in this article are solely those of the authors and do not necessarily represent those of their affiliated organizations, or those of the publisher, the editors and the reviewers. Any product that may be evaluated in this article, or claim that may be made by its manufacturer, is not guaranteed or endorsed by the publisher.
